# Chlorogenic acid isomers directly interact with Keap 1-Nrf2 signaling in Caco-2 cells

**DOI:** 10.1007/s11010-019-03516-9

**Published:** 2019-03-20

**Authors:** Ningjian Liang, John H. Dupuis, Rickey Y. Yada, David D. Kitts

**Affiliations:** Food, Nutrition, and Health Program, Faculty of Land and Food Systems, The University of British Columbia, 2205 East Mall, Vancouver, BC V6T 1Z4 Canada

**Keywords:** Chlorogenic acid isomer, Nrf2, Oxidative stress, Simulation

## Abstract

Chlorogenic acid (CGA) exists as multiple isomers (e.g., 3-CQA, 4-CQA, 5-CQA, 3,4-diCQA, 3,5-diCQA, and 4,5-diCQA) in foods such as coffee beverages, fruits and vegetables. This study aimed to investigate relative activities of these six different CGA isomers to modify redox biology in inflamed Caco-2 cells that involved Nrf2 signaling. Caco-2 cells were pre-treated with individual CGA isomers to assess the relative effectiveness to mitigate oxidative stress. Isomer-specific capacity of different CGA isomers for direct free radical scavenging activity and potential endogenous control of oxidative stress were determined using chemical assays and cell-based experiments, respectively. Molecular dynamics simulations of the CGA and Keap1-Nrf2 complex were performed to predict CGA structure-specific interactions. Results demonstrated that dicaffeoylquinic acid (diCQA including 3,4-diCQA, 3,5-diCQA, and 4,5-diCQA) isomers had greater (*p* < 0.05) affinity to ameliorate oxidative stress through direct free radical scavenging activity. This observation corresponded to greater (*p* < 0.05) capacity to activate Nrf2 signaling compared to caffeoylquinic acid (CQA including 3-CQA, 4-CQA, and 5-CQA) isomers in inflamed differentiated Caco-2 cells. Simulations revealed that differences between the ability of CQA and diCQA to interact with the Keap1-Nrf2 complex may be due to differences in relative orientation within this complex. The observed CGA isomer-specific affinity for CQA to activate Nrf2 signaling was confirmed by nuclear translocation of Nrf2 induced by CGA and greater (*p* < 0.05) upregulation of genes related to Nrf2 expression.

**Electronic supplementary material:**

The online version of this article (10.1007/s11010-019-03516-9) contains supplementary material, which is available to authorized users.

## Introduction

Chlorogenic acids (CGAs) are phenolic acids with vicinal hydroxyl groups present on aromatic residues that are derived from esterification of cinnamic acids (including caffeic acid, ferulic acid, and *p*-coumaric acid) and quinic acid [1, 2]. The ester formed between one molecule of caffeic acid and one molecular of quinic acid is called caffeoylquinic acid (CQA). There are three isomers within the CQA subgroup, including 3-CQA, 4-CQA, and 5-CQA. The ester formed between two molecules of caffeic acid, and one molecular quinic acid is called dicaffeoylquinic acid (diCQA); 3,4-diCQA, 3,5-diCQA, and 4,5-diCQA are isomers within the diCQA subgroup that are commonly present in many plant foods [3]. CGAs are reported to have bioactivities that include antioxidant, anti-inflammatory [4], and anti-colon cancer [5] activities. As a direct-acting antioxidant, CGAs interact with both reactive oxygen species (ROS) and reactive nitrogen species (RNS) by donating hydrogen atoms to reactive molecules to transform them to less active radicals [6–8]. Other studies have also indicated that CGAs can enhance production of hydroxyl radicals via the Fenton reaction [9]. Recent studies indicate that such apparent conflicting conclusions derived from former studies actually reflect the important dual roles of CGAs in maintaining an optimal cellular oxidative balance. First, CGAs elicit the beneficial effect of ROS generation to stimulate endogenous cellular signaling, which is needed in response to cell injury. Secondly, the CGAs may act to prevent excessive generation of free radicals within the cell, important because without coupling with endogenous ROS control, subsequent biological damage of important cellular constituents will result [10]. A complete understanding of this complex multifunctional affinity to control redox balance in intestinal cells is still in question.

NF-E2-relaed Factor 2 (Nrf2) is a master regulator of cellular resistance to oxidative stress that works to promote expression of cytoprotective genes in response to cellular oxidative stress [11]. Kelch-like ECH-associate protein 1 (Keap1) is a substrate adaptor component of a Cul3-dependent E3 ubiquitin ligase complex that represses activity of the transcriptional factor Nrf2 and contributes to the rapid degradation of Nrf2 [12]. There are examples of free radicals that stimulate signaling leading to Keap1 cysteine thiol residues modification, altered Keap1-E3 ubiquitin ligase activity, both of which leading to the stabilization and activation of Nrf2. Upon experiencing oxidative stress, Nrf2 is released from Keap1 repression, translocated to the nucleus, and activates the transcription of antioxidant enzyme genes [13]. This event represents an important endogenous control of oxidative stress. The relative capacity of different CGA isomers to trigger underlying Nrf2 activity and associated mechanisms that translate to maintaining oxidative balance remains to be shown.

The human intestinal Caco-2 cell line has been widely used to model the intestinal barrier and factors that influence bioavailability of substrates. Culturing the Caco-2 cells for up to 21 days transforms it to form a monolayer of cells that express several morphological and functional characteristics of the mature enterocyte [14]. Our laboratory has used a mixture of human interferon γ (IFNγ) and phorbol myristate acetate (PMA) to model an inflammatory state in the differentiated Caco-2 cell line [15]. ROS are also implicated in the development of inflammation in this model. Former studies reported from our laboratory demonstrated activation of Nrf2 signaling in response to the INFγ and PMA challenge [16] which in turn was modified by presence of a dietary source of antioxidant.

In the present study, we used an inflamed Caco-2 cell model, pretreated with different CGA isomers, to assess the relative effectiveness of these dietary polyphenols in responding to oxidative stress. Initial experiments were conducted to determine isomer-specific capacity of different CGAs for direct free radical scavenging activity using chemical assays, which were followed up with cell-based experiments to test relative potential for endogenous control of oxidative stress. We designed experiments to confirm relative affinities of six different CGA isomers to influence redox biology in inflamed Caco-2 cells that involved Nrf2 signaling. Our experimental hypothesis is that CGAs are isomer specific to ameliorate intestinal oxidative stress through direct free radical scavenging activity that is related to activation of Nrf2 signaling. Molecular dynamics (MD) simulations were utilized to assist in deconvolution of isomer-specific interactions by providing an atom-resolution view of the associations between CGA and the Keap1/Nrf2 complex. A mechanism for the activation of Keap1/Nrf2 complex by the direct free radical scavenging capacity of CGAs is proposed.

## Methods

### Materials and reagents

Human interferon γ (IFNγ), 3-(4,5-dimethylthiazol-2-Yl)-2,5-diphenyltetrazolium bromide (MTT), modified Eagle’s medium (MEM), phorbol 12-myristate 13-acetate (PMA), sodium dodecyl sulfate (SDS), 2,2′-azobis(2-amidinopropane) dihydrochloride (AAPH), dichlorofluorescein diacetate (DCFH-DA), Hanks’ balanced salt solution (HBSS), bovine serum albumin (BSA), paraformaldehyde, Triton™ X-100 and 4′,6-diamidino-2-phenylindole (DAPI) were purchased from Sigma (St. Louis, MO, USA). Chlorogenic acid isomers (3-CQA, 4-CQA, 5-CQA, 3,4-diCQA, 3,5-diCQA, and 4,5-diCQA) were obtained from Cerillian Corporation (Round Rock, TX, USA) and Chengdu Must Bio-Technology Co. (Chengdu, Sichuan, China). Penicillin, streptomycin, and FBS were purchased from Gibco® (Grand Island, NY, USA). Human IL-8 Single Analyte ELISA kits were purchased from Qiagen (Hilden, Germany).

### 2, 2′-Azino-bis-3-ethylbenzothiazoline-6-sulfonic acid (ABTS) assays

The ABTS assays were applied as described with modifications [17, 18]. Radical ABTS cations were generated by mixing 7 mM ABTS with 2.45 mM potassium persulfate in distilled water. Concentrations of Trolox (0–0.25 mM) or appropriate concentrations of CGA isomers (0–0.2 mM) were mixed with ABTS working solution and incubated at room temperature for 10 min. The absorbance was measured at 734 nm. Ratios between the slopes of the regression equations for CGA isomers and Trolox were defined as coffee antioxidant capacity. The antioxidant activity of CGA isomers is expressed as mmol Trolox equivalents/mmol of sample.

### ORAC assays

The antioxidant activities of coffee brew extracts were assessed using oxygen radical absorbance capacity fluorescein (ORAC) assays [19] and from ratios between regression equations slopes for CGA isomers and Trolox. Trolox standard (final concentrations of 0–6.0 mM) or appropriate concentrations of CGA isomer (0–1 mM) were mixed with 60 µl fluorescein (Sigma, St. Louis, MO, USA) (final concentration 60 nM) and were incubated at 37 °C for 10 min. This was followed by adding 60 µl AAPH (final concentration 12 mM), and the quench of fluorescence was monitored every minute for 60 min at the excitation and emission wavelength of 485 nm and 527 nm, respectively. The area under the fluorescence decay curve (AUC) was used as indication of the retention of fluorescein. The regression equation between the concentration of Trolox/CGA isomer and the AUC was calculated, and the ratio between the slope of coffee regression equation to the slope of Trolox regression equation was used to express the antioxidant activity of the CGA isomer. All of the analyses were run in triplicate. The peroxyl radical scavenging capacities (ORAC value) of coffee bean extracts were expressed as mmol Trolox/mmol of CGA isomer.

### Nitric oxide assays

The ability of coffee brew extracts to scavenge nitric oxide radicals (NO⋅) was determined as described previously [20]. Briefly, sodium nitroprusside (5 mM) in phosphate-buffered saline (pH 7.4) was mixed with Trolox (0–0.1 mM) or CGA isomers (0–0.02 mM) at different concentrations and incubated at 25 °C for 150 min. The amount of NO⋅ produced was assayed by measuring nitrite accumulation using a microplate assay method based on the Griess reaction. The ratio between regression equation slopes for CGA isomers and for Trolox was defined as nitric oxide radical-scavenging ability and is expressed as mmol equivalents of Trolox/mmol of CGA isomer.

### Cell culture

The human colon adenocarcinoma cell line, Caco-2 (HTB-37, American Type Culture Collection, Manassas, VA, USA), was cultured in complete MEM containing Earle’s salts supplemented with 10% FBS (Thermo Fisher Scientific Inc., Waltham, MA, USA), 100 U/mL penicillin, and 100 µg/mL streptomycin. Briefly, cells (passages 26–37) were maintained in 75–cm^2^ plates (Corning Inc., Corning, NY, USA) at 37 °C in a 5% CO_2_ humidified atmosphere, with media changes every 2–3 days. Cultured cells were split (1:5) using 0.25% (w/v) trypsin in 0.53 mM ethylenediaminetetra-acetic acid (EDTA) (Thermo Fisher Scientific Inc.) upon reaching ~ 80% confluence. Cells (1 × 10^5^/cm^2^) were seeded for individual experiments into 6- or 96-well plates (Sarstedt AG & Co., Sarstedtstrabe, Numbrecht, Germany) and cultured for 21 days with media changes every 2–3 days to elicit spontaneous differentiation.

### Assessment of cell viability

Cellular metabolic activity was assessed using MTT assays as an indirect measure of viability. Cells were incubated with test sample in 96-well plates, rinsed with phosphate-buffered saline (PBS) and incubated with serum-free medium containing 0.5 mg/mL MTT for 4 h in the dark at 37 °C. Formazan crystals were dissolved by incubating the cells with SDS (10% w/v) in 0.1 M HCl for 12 h. Amounts of formazan in wells were determined spectrophotometrically by measuring absorbance at 540 nm using a plate reader (Thermo LabSystems, Beverley, MA, USA). Cell viability (% control) was calculated from the equation:

$${\text{Viability}}\,{\text{~}}\left( {{\text{\% }}\,{\text{of~}}\,{\text{control}}} \right)=\frac{{{\text{~A}}{{\text{b}}_{{\text{sample}}}}}}{{{\text{A}}{{\text{b}}_{{\text{negative}}\,{\text{~control}}}}}}\times 100{\text{\% }}$$

where Ab_sample_ and Ab_negative control_ represent the absorbance of cells incubated with test samples and only medium, respectively.

### Ability of CGA isomers to scavenge peroxyl radicals induced by AAPH in Caco-2 cells

The effects of CGA isomers on intracellular oxidation initiated by peroxyl radicals were assessed as described previously [21]. Caco-2 cells (1 × 10^5^/mL) seeded in 96-well plates were incubated for 21 days to elicit differentiation. The cells were then incubated with medium alone (control) or with individual CGA isomers at concentrations of 0.2, 1, or 2 mM in medium at 37 °C for 24 h. The cells were rinsed with PBS (pH 7.2) and incubated with a DCFH-DA probe (5 µM) in PBS at 37 °C for 30 min. Intracellular oxidation was initiated by rinsing the cells with fresh PBS, then adding 100 µL of HBSS containing 1 mM AAPH. Fluorescence emission was measured using a Fluoroskan Ascent™ FL luminometer (Thermo Fisher Scientific, Waltham, MA, USA) at excitation and emission wavelengths of 485 and 527 nm, respectively, one hour after adding AAPH. Results are expressed as:

$${\text{\% ~}}\,{\text{Fluorescence~}}\,{\text{Inhibition}}=\frac{{{{\text{F}}_{{\text{pc}}}} - {{\text{F}}_{{\text{CGA}}}}}}{{{{\text{F}}_{{\text{pc}}}} - {{\text{F}}_{{\text{nc}}}}}}\times100{\text{\% }}$$

where F_pc_, F_nc_, and F_CGA_ represent the fluorescence emission intensity of cells incubated with the DCFH-DA probe followed by AAPH, the DCFH-DA probe alone, and individual CGA isomers followed sequentially with the DCFH-DA probe and AAPH.

### Cell challenge with PMA + IFNγ

Caco-2 cells that were cultured to differentiation were incubated for 24 h with serum-free MEM containing CGA isomers that had been previously sterilized by passage through a 2-µm filter. The medium was then replaced with fresh serum-free MEM, and the cells were incubated with 8000 U/mL IFNγ and 0.1 µg/mL of PMA (PMA + IFNγ), with or without (control) fresh CGA isomers for various periods. Blanks comprised Caco-2 cells incubated without either CGA isomers or PMA + IFNγ.

### Effects of CGA isomers on Nrf2 signaling in Caco-2 cells challenged with PMA + IFNγ

Nrf2 binding to antioxidant/electrophile response element (ARE) consensus binding sites was evaluated using Nrf2 Transcription Factor Assay Kits (600590, Cayman Chemical Company, Ann Arbor, MI, USA) to determine the role of CGA isomers in activating Nrf2 signaling in Caco-2 cells challenged with IFNγ + PMA. In brief, Caco-2 cells were scraped from plates into ice-cold PBS containing phosphatase inhibitors. Cytosolic and nuclear fractions were separated using Nuclear Extraction Kits (10009277, Cayman Chemical Company, Ann Arbor, MI, USA) according to the manufacturer’s instructions. Protein concentrations were determined using bicinchoninic acid (BCA), and then, all samples were standardized to 2 mg/mL. Portions (10 µL) containing 20 µg of cellular protein extract were incubated with immobilized oligonucleotides harboring ARE consensus binding sites (5′-GTCACAGTACTCAGCAGAATCTG-3′). The active form of Nrf2 that bound to the oligonucleotides was detected using anti-Nrf2 primary antibody (1:1000) for 1 h, followed by incubation with horseradish peroxidase (HRP)-conjugated secondary antibody (1:1000) for 1 h at room temperature. Absorbance at 450 nm was determined using a microplate reader. A representative Western blot showing the Nrf2 response of Caco-2 cells to CGA isomers is presented in Supplement Fig. 1.

### Nrf2 nuclear translocation immunocytochemistry

Caco-2 cells were first washed three times with PBS; then, the cells were fixed on slides with ice-cold 2% paraformaldehyde for 20 min, then incubated with 0.1% Triton™ X-100 for 10 min and washed three times with PBS. Non-specific binding was blocked with 3% BSA in PBS at room temperature for 30 min, and then, the cells were incubated with diluted (1:1000) anti-Nrf2 polyclonal primary antibody (Abcam Inc., Cambridge, UK) at 4 °C, overnight. After four washes with PBS, the cells were incubated with goat anti-rabbit FITC IgG (1:1000) (Thermo Fisher Scientific Inc.) for 1 h. After an additional wash with PBS, nuclei were labelled using fluorescence mounting medium containing 1 µg/mL of DAPI. Immunofluorescent signals were visualized using a Zeiss fluorescence microscope (Zeiss Group, Oberkochen, Germany).

### Real-time PCR microarrays

Total RNA was isolated from Caco-2 cells using RNeasy Mini kits (Qiagen, Valencia, CA, USA). Contaminating DNA was removed from isolated RNA using RT1 First Strand Kits (Qiagen, Valencia, CA, USA); then, RNA was transcribed to cDNA using RT2 Reaction Ready First Strand Synthesis Kit (Qiagen) and analyzed using a RT2 Profiler PCR array (Cat. no. CLAH23927, Qiagen) that was custom-designed to focus on gene families associated with oxidative stress responses. Supplementary Table S1 lists details of the array. Each experiment comprised three independent replicates of Caco-2 cells incubated with CGA isomers and challenged with IFNγ + PMA for 4 or 8 h. The negative and positive controls comprised cells incubated without and with PMA + IFNγ, respectively, for various periods. Reverse transcribed, genomic DNA, and positive PCR controls were also incubated with CGA isomers. Amplification of cDNA by PCR proceeded as described by the manufacturer using the CFX96 Touch™ Real-Time PCR Detection System (Bio-Rad Laboratories, Hercules, CA, USA) and RT2 SYBR Green qPCR Mastermix (Qiagen, Valencia, CA, USA) under the following conditions: 95 °C for 10 min, 40 cycles at 60 °C for 60 s and 95 °C for 15 s, with a melting curve from 60 to 95 °C to ensure the amplification of a single product. Data were normalized using the housekeeping genes β-actin and glyceraldehyde-3-phosphate dehydrogenase (GAPDH), then analyzed by comparison with 2^(−ΔΔCt)^ of the normalized sample^27^, were ΔΔCt = (Ct, _Target gene in treatment group_ − Ct, _Internal control gene in treatment group_) − (Ct, _Target gene in control group_ − Ct, _Internal control gene in control group_). A minimum twofold increase or decrease in gene expression compared with control was regarded as significant. The complete gene array is presented in Supplementary Table 1.

### Computational methods

All simulations were completed using the Gromacs 5.0.4 molecular dynamics (MD) simulation package and the CHARMM36 forcefield [22, 23]. CHARMM forcefield-compatible parameters for CGA isomers were generated using ParamChem and CHARMM General Forcefield (CGenFF) parameters [24, 25]. All bonds were constrained using the LINear Constraint Solver (LINCS) algorithm and Coulomb and van der Waals interactions were both calculated using the Particle Mesh Ewald (PME) algorithm [26, 27] with a potential that smoothly shifted to zero between 0.9 and 1.0 nm. Human NRF2- Kelch-like ECH-associated protein 1 (KEAP1) protein complex was obtained from the Protein Data Bank (PDB ID 2FLU) [28]. As the structure of the protein complex is a truncated form of the full sequence, the N and C termini were capped with acetyl and amide groups, respectively. The structure was placed in a simulation cell with periodic boundaries in the XYZ dimensions and solvated in TIP3P water with ions added for neutralization and to reduce the NaCl concentration to 150 mM. The resulting structure was energy-minimized and equilibrated in two rounds using a 2 fs time step: 500 ps isothermal-isochoric (NVT) at 310 K (0.1 ps coupling constant) followed by 500 ps in an isothermal-isobaric (NPT) ensemble at 310 K and 1 atm (2 ps coupling constant). After simulation for 100 ns, the final protein coordinates were docked with each of the six CGA isomers using the Swiss Dock webserver to generate potential starting configurations [29]. The docked structure with the lowest estimated Gibbs free energy was selected as the starting configuration. Each of these docked structures was solvated and had ions added to 150 mM NaCl, followed by the same minimization, and equilibration procedures were applied as described before unrestrained MD for 100 ns. All analyses of the final 20 ns of trajectories were completed using built-in Gromacs analysis tools.

### Statistics

All experimental conditions proceeded in triplicate and were repeated at least three times. Representative data analyzed by one-way ANOVA using GraphPad Prism software (San Diego, CA, USA) are shown as means ± standard deviation (SD). Significant differences were compared using Bonferroni post hoc tests with *p* < 0.05 representing a statistically significant difference.

## Results

### Free radical scavenging capacity of CGA isomers

Table 1 shows the capacity of the six CGA isomers to scavenge ABTS⋅^+^, nitric oxide and peroxyl radicals using standard chemical assays. Dicaffeoylquinic acids generally had significantly more antioxidant activity than caffeoylquinic acids (*p* < 0.05), due to having two more hydroxyl groups in a phenolic moiety than caffeoylquinic acids. Peroxyl radical scavenging capacity was significantly differed among dicaffeoylquinic acid isomers in ORAC assays (*p* < 0.05).

**Table 1:** Antioxidant activity of CGA isomers determined using ABTS, NO radical scavenging, and ORAC assays

CGA isomers	Antioxidant activity (mmol Trolox equivalents/mmol)		
	ABTS	NO radical scavenging	ORAC
3-CQA	0.97 ± 0.02^a^	6.62 ± 0.25^a^	3.44 ± 0.09^a^
5-CQA	0.88 ± 0.04^b^	6.24 ± 0.43^a^	3.45 ± 0.06^a^
4-CQA	1.01 ± 0.01^a^	6.04 ± 0.97^a^	3.47 ± 0.09^a^
3,5-diCQA	1.88 ± 0.04^ce^	10.10 ± 0.49^b^	5.07 ± 0.14^b^
3,4-diCQA	1.96 ± 0.02^cd^	9.23 ± 0.36^b^	5.49 ± 0.15^c^
4,5-diCQA	1.86 ± 0.04^e^	10.18 ± 0.58^b^	4.53 ± 0.12^d^

Values represent means ± standard deviation of triplicate assays. Superscript letters indicate differences in antioxidant values of CGA isomers within ABTS, NO radical scavenging and ORAC assays, respectively, identified by one-way ANOVA and Bonferroni post hoc analysis at *p* < 0.05

### Capacity of CGA isomers to scavenge AAPH-generated peroxyl radicals in Caco-2 cells

**Fig. 1:**
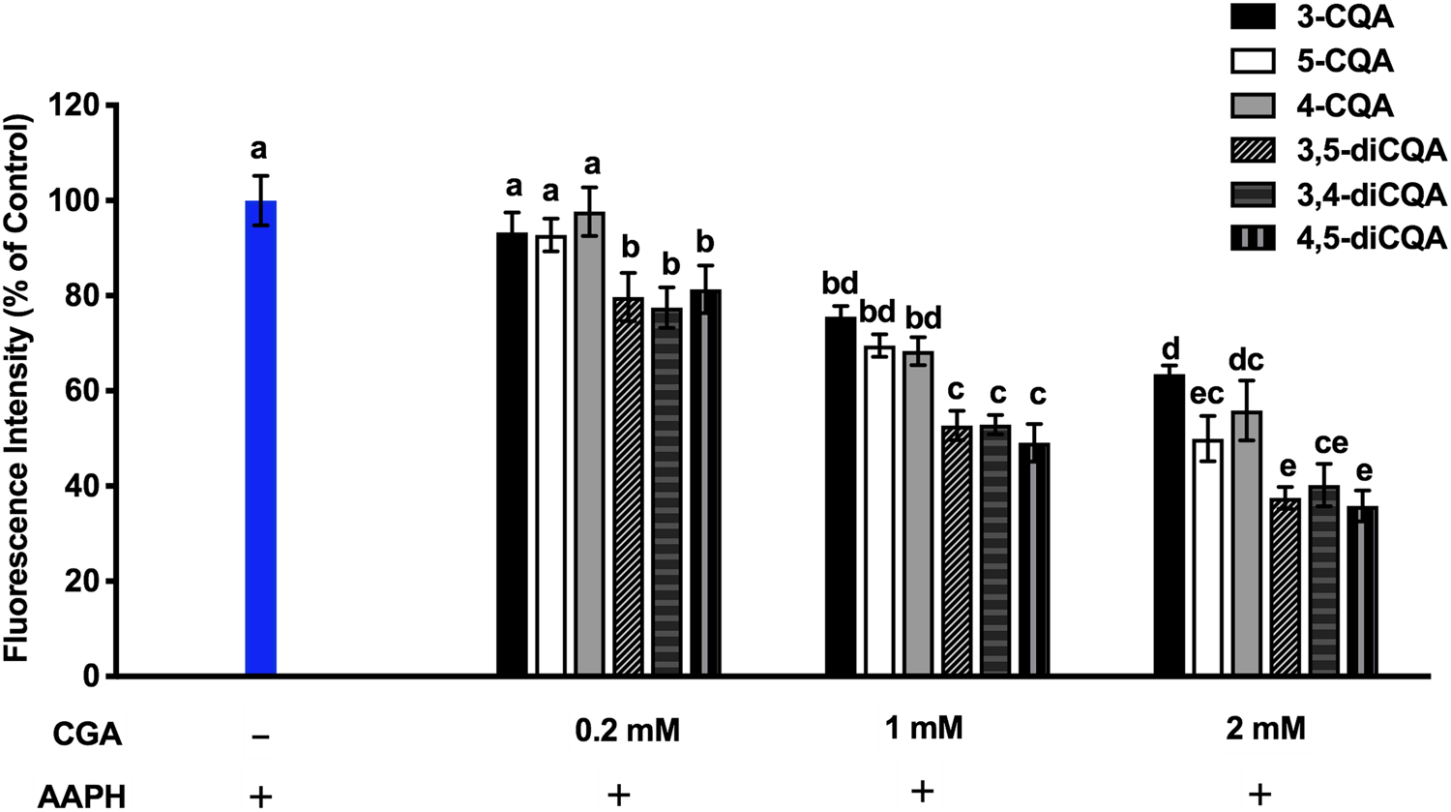
Capacity of 0.2, 1, and 2 mM CGA isomers to scavenge AAPH generated peroxyl radicals in Caco-2 cells, determined using DCFH-DA. Experiments were performed in triplicate (*n* = 3), and results were expressed as mean ± standard deviation. Differences between treatments were analyzed by one-way analysis of variance (ANOVA), using Bonferroni posttest, using GraphPad Prism software. The blue bar represents the control (without CGA, with AAPH). Superscript with different letters (a, b, c, d, or e) denotes a significant difference (*p* < 0.05)

Chemical assays such as ABTS, nitric oxide, and ORAC measure only the physiochemical radical exchange and cannot be completely translated to in vivo activity. Subsequent experiments, therefore, challenged model Caco-2 cells with AAPH in vitro to determine the antioxidant capacity of CGA isomers for scavenging peroxyl radicals. Cell viability was evaluated before conducting cellular biochemistry analysis. The decomposition of AAPH forms one and two moles of nitrogen and carbon radicals, respectively, which react with molecular oxygen to generate peroxyl radicals [30]. Figure 1 shows that the intracellular peroxyl radical scavenging capacity of CGA toward intracellular peroxyl radicals in Caco-2 cells incubated with AAPH was concentration dependent. Moreover, the ROS scavenging capacity was relatively higher for dicaffeoylquinic, than caffeoylquinic acids (*p* < 0.05). At the highest concentration tested (2 mM), 3-CQA, 4-CQA, and 5-CQA reduced 35% of ROS generated by AAPH, whereas 3,5-diCQA, 4,5-diCQA, and 3,4-diCQA reduced about 60% of ROS. Additional differences among individual caffeoylquinic and dicaffeoylquinic acid isomers, respectively, to display free radical scavenging capacity were not observed.

### Isomers of CGA upregulated Keap1-Nrf2/ARE signaling pathway in Caco-2 cells challenged with IFNγ + PMA

**Fig. 2:**
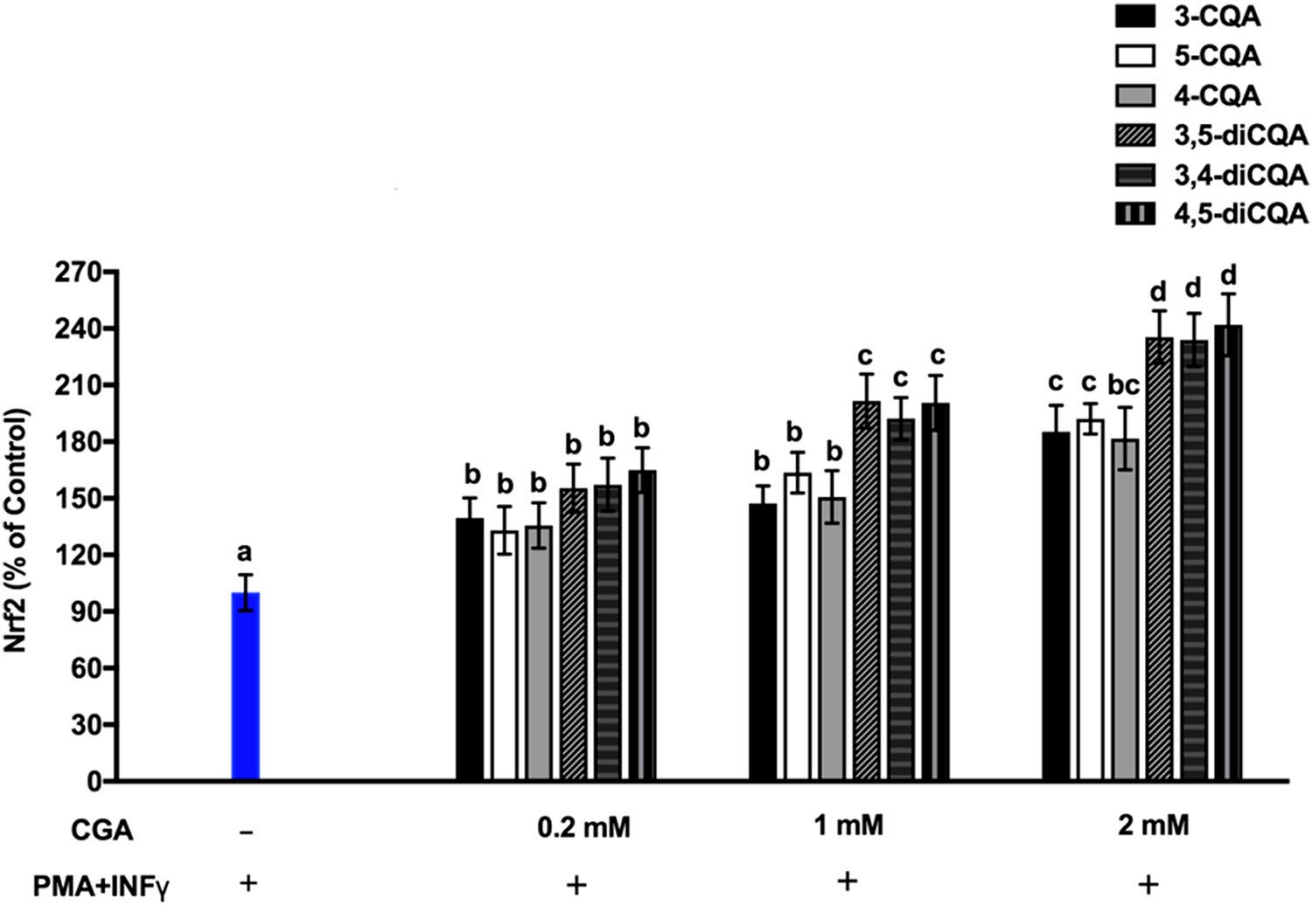
Effects of CGA isomers on Nrf2 nuclear translocation (% of Control) in Caco-2 cells induced by PMA + IFNγ. Experiments were performed in triplicate (*n* = 3), and results were expressed as mean ± standard deviation. Differences between treatments were analyzed by one-way analysis of variance (ANOVA), using Bonferroni posttest, using GraphPad Prism software. The blue bar represents the control (without CGA, with AAPH). Superscript with different letters (a, b, c, or d) denotes a significant difference (*p* < 0.05). Refer to Supplementary Fig. 1 for representative Western blot gel showing effect of different CGA isomers on nuclear Nrf2 in Caco-2 cells

**Fig. 3:**
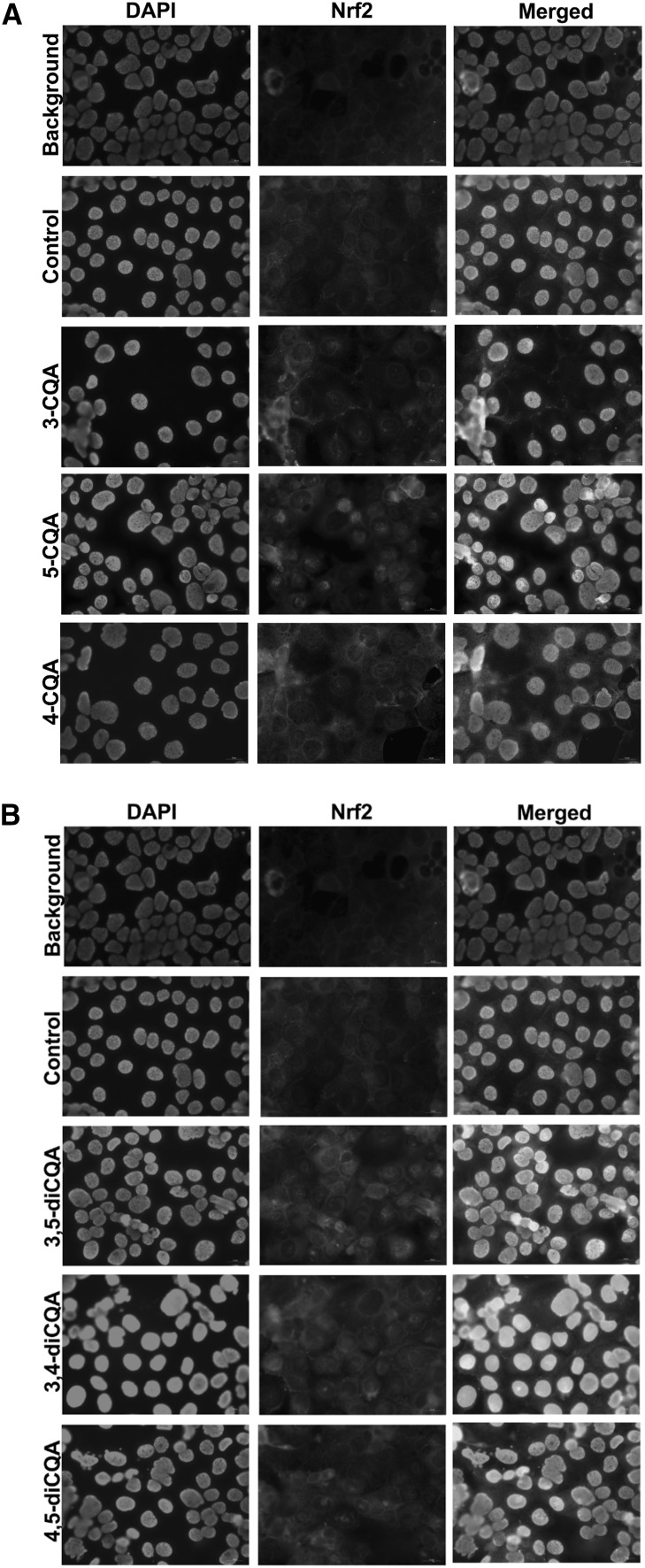
Immunocytochemistry of cells showing different individual CGA isomers at 0.2 mM enhanced Nrf2 localization into nucleus in Caco-2 cells. **a** 3-CQA, 5-CQA, and 4-CQA. **b** 3,5-diCQA, 3,4-diCQA, and 4,5-diCQA Localization of Nrf2 was performed by double immunofluorescence staining in cells with only PMA + IFNγ treatment, cells with individual CGA isomer treatment before PMA + IFNγ challenge. Background represents cells treated with PMA + IFNγ but without a primary antibody during the immunofluorescence staining. The control represents cells treated with PMA + IFNγ, and with both primary and secondary antibodies during the immunofluorescence staining. Nrf2 protein were stained in green; nuclei were stained with DAPI (blue). The merged image showed the nuclear location of Nrf2 in nuclei. (Color figure online)

The effect of CGA isomers and IFNγ + PMA treatment on the cell viability of Caco-2 cells was evaluated. With more than 85% of cell viability observed relative to unexposed cells suggests that the treatment was not cytotoxic to the Caco-2 cells. In addition to showing dietary polyphenols to neutralize ROS through direct scavenging activity, the affinity to trigger endogenous antioxidant capacity and prevent over production of ROS was also examined as evidence for maintaining intestinal homeostasis. The Keap1-Nrf2/ARE signaling pathway is essential for regulating antioxidant responses and modulating the expression of numerous antioxidant enzymes, including Glutamate-Cysteine ligase catalytic subunit (GCLC), Glutamate-Cysteine Ligase modifier (GCLM) subunit superoxide dismutase-1 (SOD)1, glutathione synthetase (GSS) and glutathione disulfide reductase (GSR). Thus, we investigated the effects of CGA isomers on the Nrf2/ARE signaling pathway in Caco-2 cells that were treated with pro-inflammatory proteins. Under normal conditions, Nrf2 is located in cytoplasm as an inactive complex with repressor Keap1, whereas stimulation by inducers such as ROS causes Nrf2 to dissociate from Keap1 and translocate to the nucleus, where it binds to antioxidant response elements located in the regulatory regions of phase II antioxidant defense genes. *Western blotting was used to measure amounts of Nrf2 protein that translocated to the nucleus to determine the functional effects of CGA isomers on the Nrf2 pathway*. Figure 2 shows that the CGA isomers concentration dependently increased ARE binding activity. At 2 mM, 3-CQA, 4-CQA, and 5-CQA increased Nrf2 binding activity ~ 1.5-fold, and 2 mM 3,4-diCQA, 4,5-diCQA, and 3,5-diCQA increased the activity 2.4-fold compared with the control. Confocal microscopy also confirmed the nuclear translocation of Nrf2 induced by CGA isomers (Fig. 3a, b). These results collectively lead us to conclude that some degree of cellular bioavailability of CGA isomers had occurred to initiate nuclear translocation of Nrf2 .

### Interactions between CGA isomers and Keap1/Nrf2 complex

**Fig. 4:**
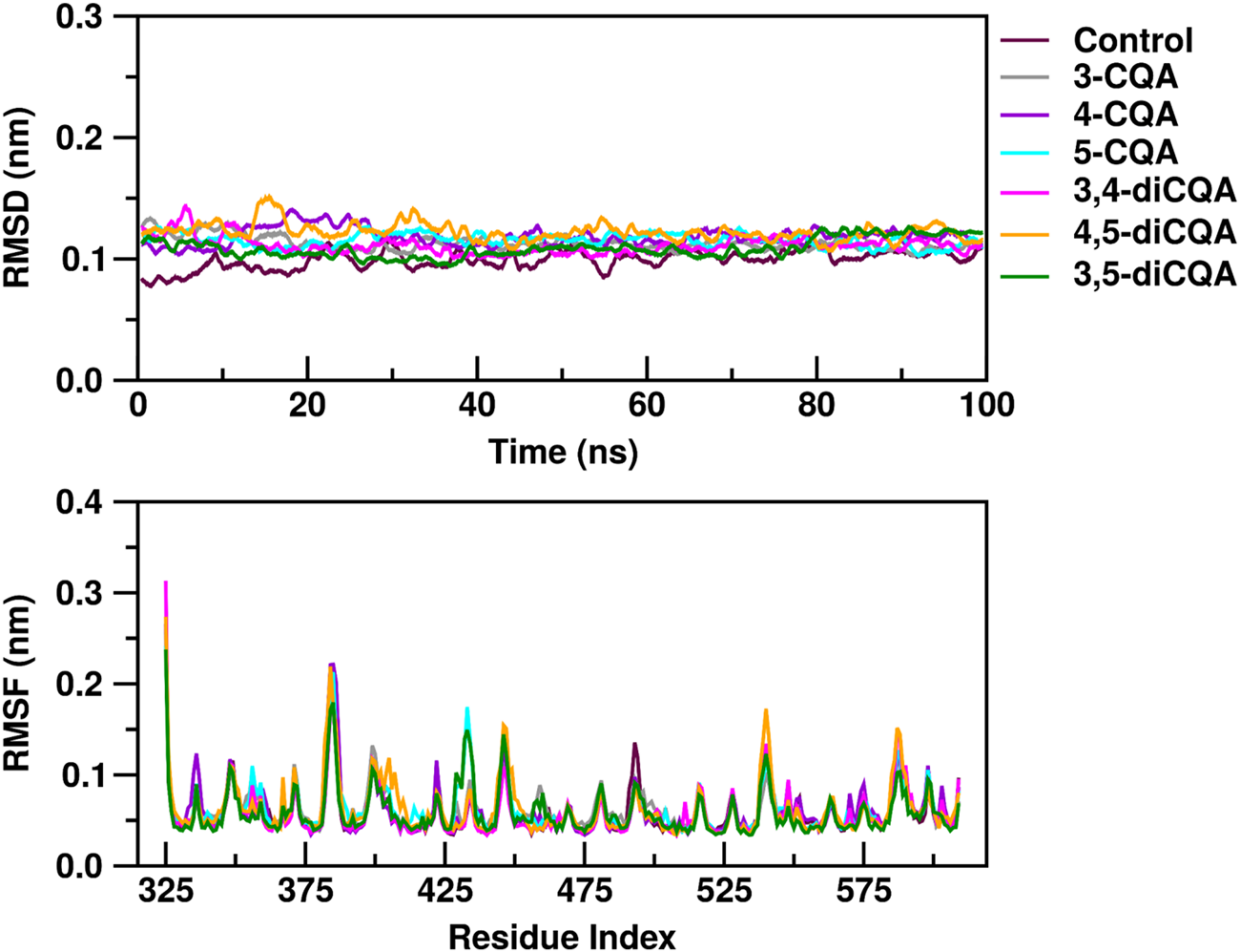
RMSD (top), and RMSF (bottom), of β-propeller of Keap1 in Keap1/Nrf2 complex containing CGA isomers. RMSD represents “root-mean-square deviation,” and RMSF represents “root-mean-square fluctuation

Interactions between the six CGA isomers and complexes of the Keap1 β-propeller with Nrf2 were studied using molecular dynamics. As the Keap1 structure equilibrated around the inserted CGA isomer, its global structure slightly changed compared with the control, and the root-mean-square deviation (RMSD, Fig. 4) was quantified using the 2FLU crystal reference. The RMSD across all simulations, including the control simulation without CGA, ranged from 0.10 to 0.12 nm; a specific mono-CQA/di-CQA effect was not found. Root-mean-square fluctuations (RMSF) were also minor relative to the control with most of the deviations attributed to the outer random coil section of the Keap1 propeller and the hydrophobic β-sheets lining the core of the Keap1 structure, which was quite stable (Fig. 4).

**Fig. 5:**
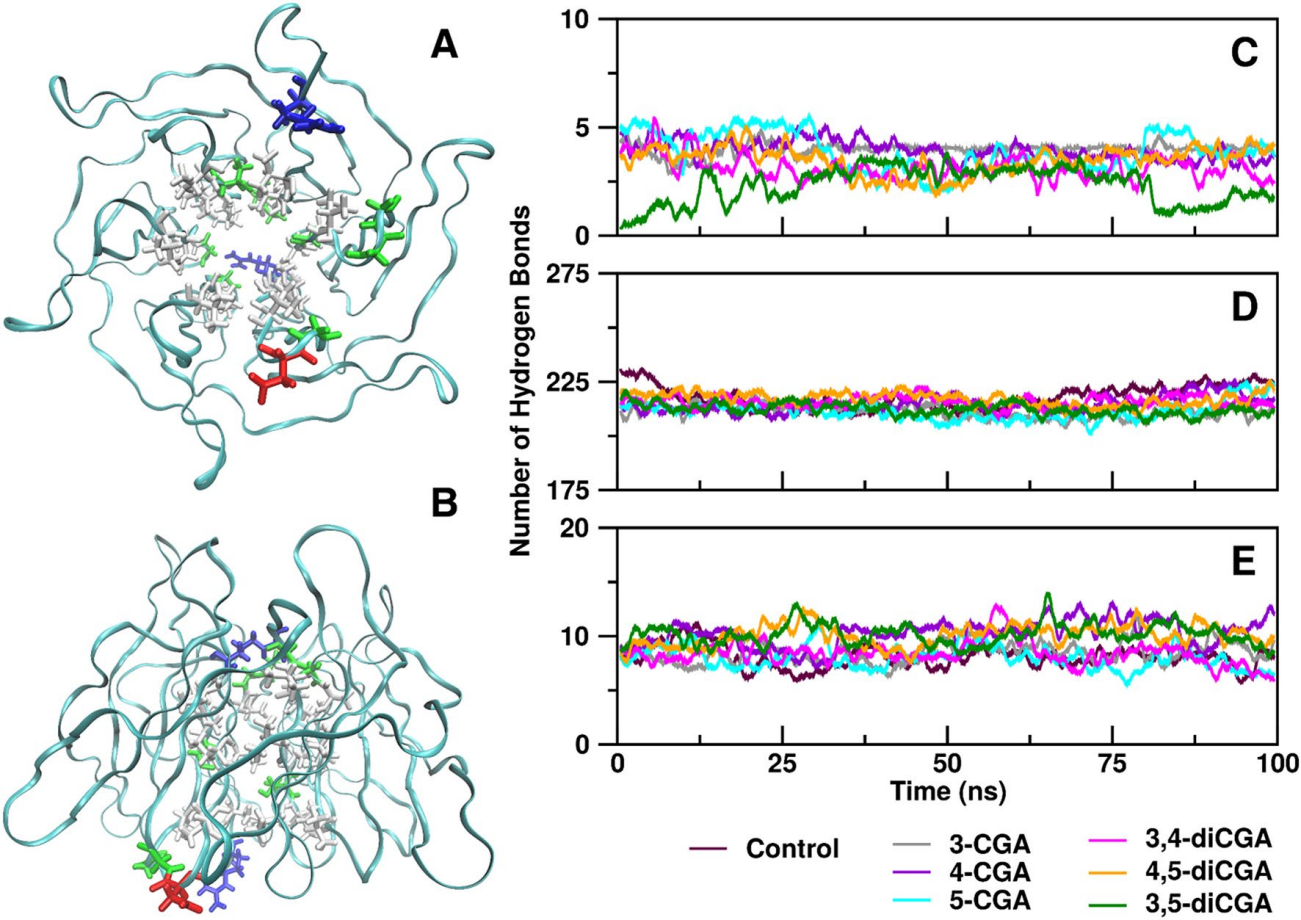
Analysis of hydrogen bonds in CGA isomers that interacted with Keap1/Nrf2 complex. Bottom (**a**) and side (**b**) views of Keap1 β-propeller according to residue types. Blue, arginine; red, aspartic acid; green, glycine, serine, or threonine; white, valine, alanine, isoleucine or leucine. Hydrogen bonds between CGA isomer and Keap1 β-propeller (**c**) and intra-Keap1 β-propeller (**d**), and between Keap1 β-propeller and Nrf2 fragment (**e**). (Color figure online)

Interactions between the CGA isomers and Keap1 were restricted to the hydrophobic core of the β-propeller and were mediated primarily through the amino (N-H) groups of valine, glycine, and isoleucine amino, and the sidechains of arginine, threonine, and serine (Fig. 5a, b). The number of intra-Keap1, CGA-Keap1, and Keap1-Nrf2 hydrogen bonds was relatively stable throughout the trajectory (Fig. 5c–e), which reflected the stability of the RMSD. The only deviation from this trend toward interaction with Keap1-CGA hydrogen bonds was for 3,5-diCQA. During the last 20 ns of the simulation, the Keap1/3,5-diCQA complex contained fewer hydrogen bonds than Keap1 and the other CGA isomers (Fig. 5c), which ranged from 3 to 4 hydrogen bonds for non-3,5-diCQA complexes. The 3,5-diCQA containing complex had an average of 1.6 hydrogen bonds; one of the caffeic acid “wings” on the 3,5-diCQA molecule became reoriented during the simulation time frame, resulting in new positions that were less amenable to forming hydrogen bonds. Hydrogen bonding between Nrf2 and the CGA isomers was undetectable. Due to the low number of Keap1-CGA hydrogen bonds, but a relatively high number of residues being involved in hydrogen bonding between these two groups (a total of 31), it was suspected that the hydrogen bonds are likely highly transient. The CGA structures are more likely to be involved in hydrophobic interactions than hydrogen bonds, especially given that most of the residues that line the core of the β-propeller are hydrophobic in nature.

**Fig. 6:**
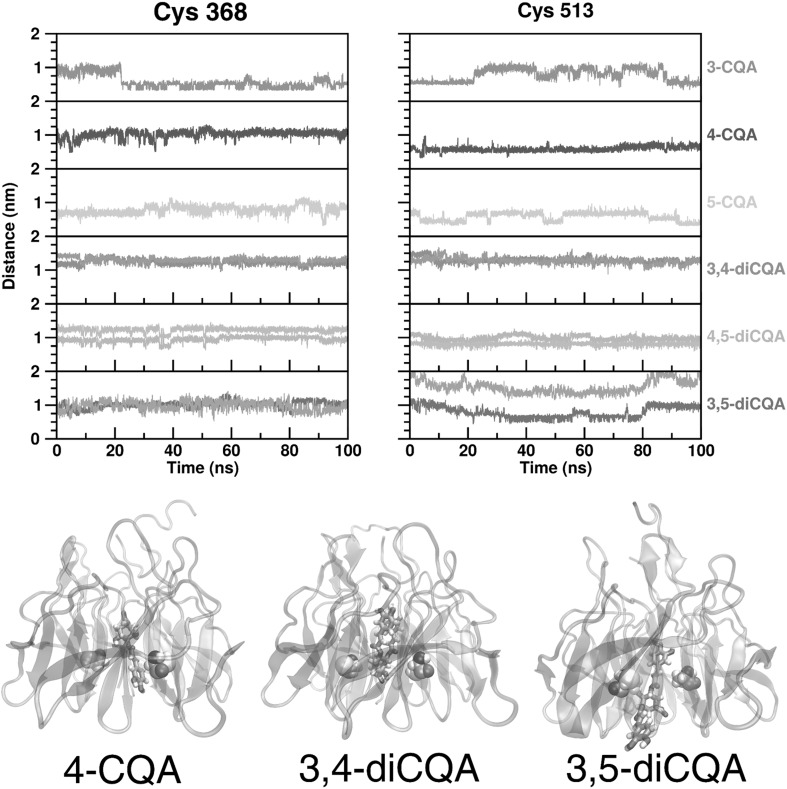
Distances between phenolic ring OH groups and S atoms of C368 or C513 and equilibrated positions of CGA isomers. Top, minimum distances between phenolic ring OH groups and S atoms in C368 or C513. Bottom, visual representation of equilibrated position of three chlorogenic acid isomers. Given structural relationships between 3-, 4-, and 5-CQA or 3, 4- and 4,5-diCQA, only one of these three were selected as representative. C368 and C513 are on left and right of all models

The proximity of hydroxyl groups on the phenolic ring to specific cysteine residues was also investigated as Keap1/Nrf2 signaling is at least partially controlled by the cysteine reduction/redox state of the sulfur atom on cysteine. The cysteine residues C368 and C513 were located near the hydrophobic core of the β-propeller where the CGA isomers preferred to reside. The minimum distance between the CGA phenolic hydroxyl groups and the S atoms of C368 and C513 was analyzed to determine whether or not the CGA isomers can protect them from oxidation (Fig. 6). The hydroxyl groups of the phenolic moieties in the diCQA isomers were separately analyzed. During the last 10 ns, the 3-, 4-, and 5-CQA isomers generally became averages of 0.54, 1.05, and 0.76 nm, respectively, closer to C368, and 0.54, 0.66, and 0.41 nm, respectively, closer to C513. The averages per phenolic group for the 3,4 and 4.5-diCQA isomers ranged from 0.81 to 1.28 nm for both C368 and C513. Lastly, 3,5-diCQA was unique in that both phenolic groups were similarly positioned relative to C368, whereas one group was, on average, 1.74 nm and the other was 0.97 nm away from C513. The discrepancies between the mono and diCQA isomers, as well as between 3,5-diCQA and the other diCQA isomers, can be explained by their orientation within the Keap1–Nrf2 complex (Fig. 6, bottom). MonoCQA isomers resided with the phenolic group oriented facing the bottom of the Keap1 β-propeller and the carbohydrate group faced upward and thus closer to the Nrf2 interface. In contrast, the 3,4 and 4,5-diCQA phenolic groups were folded on top of each other and were positioned closer to the Nrf2 interface. However, 3,5-diCQA was unique in being oriented with one phenolic group firmly within the core of the β-propeller and another outside the bottom.

### Impact of CGA isomers on gene expression

The effects of PMA + IFNγ on the expression of genes that related to Nrf2 signaling in Caco-2 cells were investigated. We assessed the relative abilities of 5-CQA and 3,5-diCQA to modulate the expression of these genes in Caco-2 cells incubated with PMA + IFNγ (Table 2). The results showed significant (*p* < 0.05) upregulation of the genes for nuclear transcription factor 2 (Nrf2), KEAP1 and nuclear factor (erythroid 2)-like 2 (NFE2L2). Corresponding to these changes, were the down-regulation of genes associated with phase II detoxification enzymes, such as glutamate-cysteine ligase catalytic subunit (GCLC), glutamate-cysteine ligase modifier subunit (GCLM), and superoxide dismutase 1 (SOD1) at 4 h. These findings confirmed that the pro-inflammatory proteins triggered a response to oxidative stress in Caco-2 cells that consequently initiated cellular antioxidant defense reactions.

**Table 2:** Effects of 5-CQA and 3,5-diCQA at Concentration of 0.2 mM on Modulation of Transcription of Genes in Redox Signaling in PMA + INFγ treated Caco-2 Cells after 4 h

Gene name	Treatments		
	PMA + INFγ	5-CQA + PMA + INFγ	3,5-diCQA + PMA + INFγ
KEAP1	2.39^a^	5.84^b^	7.22^c^
NFE2L2	2.28^a^	3.45^a^	6.42^b^
NFE2L1	1.93^a^	5.07^b^	5.88^b^
GCLC	− 9.81^a^	− 1.16^b^	5.95^c^
GCLM	− 13.00^a^	1.30^b^	4.50^c^
SOD1	− 6.19^a^	− 2.48^b^	− 1.21^b^
GPX2	2.30^a^	7.22^b^	9.34^b^
GSS	− 1.33^a^	1.10^b^	1.68^b^
GSR	− 2.08^a^	1.59^b^	1.63^b^

Values represent fold regulation, expressed as relative to untreated control cells. Different letters indicate significant (*p* < 0.05) differences observed between treatment groups analyzed by one-way ANOVA followed by Bonferroni post hoc analysis at *p* < 0.05 using GraphPad Prism. Supplementary Figure S1 lists full names and functions of genes in this table. Only the result of genes related to Nrf2 signaling is reported in this table

## Discussion

The underlying mechanisms of action between phenolic acids toward free radicals that are generated by common cellular metabolic activities will depend on differences in the molecular structures specific to antioxidant components. These chemical characteristics of the free radical under investigation and the specific radical assay method used to quantify antioxidant activity require consideration in defining the precise mechanism of action of the phenolic component. For example, the peroxyl radical used for ORAC tests interacts with CGA at hydroxyl groups attached to the carbon atom with the largest electron density in the benzene ring. This is different than the ABTS radical, which interacts with CGA depending on the degree of ionization with carboxyl and phenolic hydroxyl groups. In our preliminary experiment, the ORAC value for 5-CQA was comparable to that reported by Bakuradze et al. [31] and our ABTS value for 5-CQA confirmed the value reported by Gómez-Ruiz et al. [32]. Good agreement was obtained for relative affinities of both CQA and di-CQA to quench both ROS and RNS radicals, respectively [33].

It is generally regarded that the free radical scavenging capacity of CGA isomers depends on the availability of phenolic hydrogen atoms and the possibility that the phenoxyl radicals that are formed after hydrogen donation are stabilized [34, 35]. Caffeoylquinic and dicaffeoylquinic acids contain one and two cinnamic acid moieties, respectively, indicating that dicaffeoylquinic acid contains more phenolic hydrogen atoms. The double bond (–CH=CH–COOR) groups linked to the phenyl ring have an important role in stabilizing the radical by resonance. This structural characteristic explains why dicaffeoylquinic acid has relatively higher ROS scavenging capacity than caffeoylquinic acid. Various mechanisms have been proposed to explain the direct acting antioxidant properties of phenolic compounds. Among these, is the hydrogen atom transfer (HAT), whereby a hydrogen atom of a hydroxyl group (OH) is transferred from a phenol (ArOH) group to a free radical (X⋅) to form an unstable phenoxyl radical (ArO⋅) and an X-H molecule [36]. The phenoxyl radical exists as a transient intermediate potentially undergoing many reactions of both oxygen and carbon radicals before eventually becoming stabilized through conjugative resonance stabilization. The free radical scavenging capacity of hydroxycinnamic acids and related derivatives theoretically depends not only on the number of available hydrogen atoms, but also on the bond dissociation energy (BDE) of the hydrogen atom on the phenolic moiety. Furthermore, others have reported that secondary reactions that proceed when phenoxyl radicals are initially formed will contribute to differences in the relative reactivity of hydroxycinnamic acids and associated derivatives with free radicals [37]. In our study, 5-CQA and 3,5-diCQA which contain two and four hydroxyl groups, respectively, in phenolic moieties, displayed ORAC value for 3,5-diCQA that was not double that of 5-CQA. This suggests that the BDE of the hydrogen atoms of the phenolic moiety in 3,5-diCQA and in 5-CQA is non-linear and thus not a simple 2:1 relationship. It is therefore important that numerous molecular properties that include both the number and the location of hydroxyl groups in the aromatic ring, the location of the conjugated double bond, and the characteristics of the initially formed phenoxyl radical are all considered when describing the affinity of free radical scavenging by individual CGA isomers.

The initial comparison of CGA direct free radical scavenging activity, obtained using simple chemical methods, relative to a more complex cell-based model was conducted using the intestinal Caco-2 cell, to determine whether a similar pattern of activity could be reproduced. The concentration-dependent activity of dicaffeoylquinic acids having relatively greater ROS scavenging activity compared to caffeoylquinic acids confirmed the results obtained using chemical assays. It was also concluded that a similar degree of bioavailability existed for all CGA isomers in order to achieve relative antioxidant capacity in the cell-based model. To characterize antioxidant properties of CGAs on free radical scavenging alone, however, could lead to a biased, or oversimplified approach to understanding completely the roles and functions of molecules including phytochemicals proposed in human health and disease [38]. For example, many structurally diverse phytochemicals that affect change in oxidative stress and influence redox balance have a striking propensity to also modulate a series of cell signaling events that control cellular homeostasis by altering the structures of receptors or modulating transcription factors. In particular, two critical cysteine residues (C273 and C288) in Keap1 are required for the Keap1-dependent ubiquitination of Nrf2 [39]. Sulforaphane from broccoli is an example of an antioxidant compound that can modify Keap1 cysteine residues leading to Keap1–Nrf2 interactions to dissociate [40]. It is proposed herein that a similar mechanism for CGAs also exists where CGA isomers upregulate Nrf2 nuclear localization. A complex transduction of signaling cascade events triggered by CGA isomers in response to changes in cellular redox balance is involved in the underlying mechanism(s) of action. To achieve the latter, it is proposed that the intracellular donation of electrons by CGA isomers to free radicals results in the conversion to phenoxyl radicals (ArO⋅), which in turn subsequently directly modulate Nrf2 signaling. The chemical assays demonstrated the capacity of CGAs to scavenge free radicals, a necessary first step in the formation of transient radicals. For example, phenolic phytochemicals trap chain-carrying peroxyl radicals (ROO⋅) to form hydroperoxide (ROOH) and derived resonance-stabilized phenoxyl radicals (ArO⋅) (Reaction 1) [40]. Derived phenoxyl, or transient, radicals react with other peroxyl radicals to form non-radical products, which terminates the reaction (Reaction 2), or alternatively, which then react again to produce another peroxyl radical (Reaction 3) [41]:

1 $${\text{ROO}}^{\cdot} +{\text{ArOH}} \to {\text{ROOH}}+{\text{ArO}}^{\cdot}$$

2 $${\text{ROO}}^{\cdot} +{\text{ArO}}^{\cdot} ~ \to {\text{nonradical products}}$$

3 $${\text{ArO}}^{\cdot} +{\text{RH}} \to {\text{ArOH}}+{\text{R}}^{\cdot} \to {\text{ROO}}^{\cdot}$$

where ROO⋅, ROOH, ArOH, ArO⋅ ,RH and R⋅ represent peroxyl radicals, a mono-substituted derivative of hydrogen peroxide, phenolic acid, phenoxyl radicals, respectively. A cycle of phenoxyl radical can occur.

It is hypothesized that phenoxyl radicals generated by CGA from direct free radical scavenging have an important function to enable direct molecular contact with the cysteine residues in Keap1 to oxidize those in the Keap1-Nrf2 structure. The net effect results in activation of Nrf2 signaling. Docking experiment showed that many caffeic acid moiety hydroxyl oxygens came into close proximity to the sulfur atoms in cysteine during a 100 ns simulation. Minimum distances between these groups for C368 ranged from 3.1 to 6.2 Å for all isomers except for 3,4-diCQA, which was slightly further out at 9.4 Å. For C513 the trend was similar—the minimum distances ranged from 3.1 to 5.8 Å, again for all except for the outlier of 3,4-diCQA at 8.7 Å. Given that the van der Waals radii of oxygen and sulfur are 1.5 and 1.8 Å [42], the lower ends of these values essentially represent minimal atomic separations and indicate direct atomic contact. Given the plasticity of protein structures and the potential for alternative orientations of the isomers in vivo, CGA isomers with phenolic hydroxyl groups converted to radicals at these distances could be in direct contact with the cysteine side chains and cause oxidation of SH groups to S⋅. This could lead to the dissociation of Nrf2 from Keap1 and result in its translocation to the nucleus, where it binds to antioxidant response elements located in the regulatory regions antioxidant defense genes. Results from the present study indicated a CGA isomer specific activation of Nrf2 which lead to a significant upregulation of genes associated with Nrf2 expression, compared to Caco-2 cells challenged only with PMA + INFγ. Moreover, 3,5-diCQA displayed both greater upregulation of KEAP1 and NFE2L1 compared to 5-CQA treated cells, as well enhanced NRf2 gene expression with the additional upregulation of NFE2L2. This result was attributed to the plausible greater expression of Keap 1 and Nrf2 subunits that occur to compensate the removal of cytoplasmic Nrf2 due to the nuclear translocation. This explanation is confirmed by intracellular antioxidant test results that showed CGAs exhibit isomer specific activity toward maintaining cellular oxidative balance, which in turn was related to an initial capacity to directly scavenge free radicals, as shown in the chemical antioxidant tests.

## Electronic supplementary material

Below is the link to the electronic supplementary material.

Supplementary material 1 (DOCX 872 KB)

## Abbreviations

AAPH: 2,2′-Azobis(2-amidinopropane) dihydrochloride
CGA: Chlorogenic acid
CQA: Caffeoylquinic acid
DCFH-DA: Dichlorofluorescein diacetate
DAPI: Triton™ X-100 and 4′,6-diamidino-2-phenylindole
diCQA: Dicaffeoylquinic acid
IFNγ: Human interferonγ
Keap1: Kelch-like ECH-associate protein 1
PMA: Phorbol myristate acetate
RMSD: Root-mean-square deviation
RMSF: Root-mean-square fluctuation
ROS: Reactive oxygen species
RNS: Reactive nitrogen species
MD: Molecular dynamics
MTT: 3-(4,5-dimethylthiazol-2-Yl)-2,5-diphenyltetrazolium bromide
MEM: Modified Eagle’s medium
Nrf2: NF-E2-relaed factor 2
