# Supplementary material for: Chlorogenic acid isomers directly interact with Keap 1-Nrf2 signaling in Caco-2 cells
Source: Mol Cell Biochem. 2019 Mar 20;457(1):105–18. doi: 10.1007/s11010-019-03516-9 (PMC6548765; doi:10.1007/s11010-019-03516-9)
Supplement: Supplementary file 1 — Supplementary material 1 (DOCX 872 KB) [file 11010_2019_3516_MOESM1_ESM.docx]

**Supplementary Data.**

Table S1: List of Genes Included in Custom Real Time PCR Array

| Gene | Full name | Functions of encode protein |
| --- | --- | --- |
| HMOX1 | Heme oxygenase 1 | Catalyzes the degradation of heme |
| GCLC | glutamate-cysteine ligase catalytic subunit | first rate-limiting enzyme of glutathione synthesis |
| GCLM | glutamate-cysteine ligase modifier subunit | the first rate limiting enzyme of glutathione synthesis |
| NQO1 | NAD(P)H quinone dehydrogenase 1 | prevents the one electron reduction of quinones that results in the production of radical species |
| KEAP1 | kelch like ECH associated protein 1 | Interact with Nrf2 and is important for the amelioration of oxidative stress |
| NFE2L2 | nuclear factor, erythroid 2 like 2 | A basic leucine zipper protein that regulates the expression of antioxidant proteins that protect against oxidative damage |
| SOD1 | superoxide dismutase 1 | An enzyme that catalyzes the dismutation of the superoxide radical into oxygen or hydrogen peroxide. |
| CAT | catalase | An enzyme that catalyzes the decomposition of hydrogen peroxide to water and oxygen. |
| GPX1 | glutathione peroxidase 1 | A member of the glutathione peroxidases family that reduce lipid hydroperoxides to their corresponding alcohols and to reduce free hydrogen peroxide to water. |
| GPX2 | glutathione peroxidase 2 | A member of the glutathione peroxidases family that reduce lipid hydroperoxides to their corresponding alcohols and to reduce free hydrogen peroxide to water. |
| GPX4 | glutathione peroxidase 4 | A member of the glutathione peroxidases family that reduce lipid hydroperoxides to their corresponding alcohols and to reduce free hydrogen peroxide to water. |
| NFE2 | Transcription factor NF-E2 45 kDa subunit | Regulates the expression of [antioxidant](https://en.wikipedia.org/wiki/Antioxidant) proteins that protect against [oxidative damage](https://en.wikipedia.org/wiki/Oxidative_stress) |
| NFE2L1 | Nuclear factor erythroid 2-related factor 1 | Regulates the expression of [antioxidant](https://en.wikipedia.org/wiki/Antioxidant) proteins that protect against [oxidative damage](https://en.wikipedia.org/wiki/Oxidative_stress) |
| OXSR1 | Oxidative stress responsive 1 | Oxidative stress responsive |
| CYP1A1 | cytochrome P450 family 1 subfamily A member 1 | Involved in phase I xenobiotic and drug metabolism. |
| GSTA1 | glutathione S-transferase alpha 1 | Add glutathione to target electrophilic compounds, including carcinogens, therapeutic drugs, environmental toxins, and products of oxidative stress. |
| GSTK1 | glutathione S-transferase kappa 1 | catalyzes the conjugation of glutathione to a wide range of hydrophobic substates facilitating the removal of these compounds from cells |
| GSTO1 | glutathione S-transferase omega 1 | Glutathione-dependent thiol transferase and dehydroascorbate reductase activities. |
| GSS | Glutathione synthetase | Glutathione biosynthesis |
| GSR | Glutathione reductase | Reduction of GSSG to GSH |
| ACTB | Beta-actin | Housekeeping gene |
| GAPDH | Glyceraldehyde 3-phosphate dehydrogenase | Housekeeping gene |


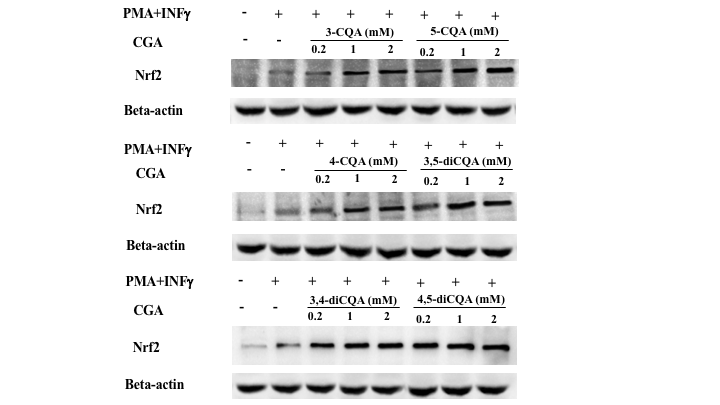


Figure S1: A representative Western Blotting gel showing the effects of different CGA isomers on the nuclear level of Nrf2 in Caco-2 cells. β-actin served as the house keeping control.
